# PROTOCOL: Home‐based care for people with dementia: A systematic review

**DOI:** 10.1002/cl2.1285

**Published:** 2022-11-02

**Authors:** Yanfei Li, Xiuxia Li, Rui Li, Nan Chen, Kehu Yang

**Affiliations:** ^1^ Evidence‐Based Medicine Center, School of Basic Medical Sciences Lanzhou University Lanzhou China; ^2^ Evidence‐Based Social Sciences Research Center/Health Technology Assessment Center, School of Public Health Lanzhou University Lanzhou China; ^3^ Research and Education Department Shanxi Provincial Rehabilitation Hospital Xi'an China

## Abstract

The abstract section should read: This is the protocol for a Campbell systematic review. The objectives are as follows: (1) Which formal HBC services for people with dementia have been tested in an RCT? (2) How effective are the different formal HBC services in improving the physical and mental health outcomes of people with dementia? (3) What is the cost and patient's health service usage of different formal HBC services?

## 1 BACKGROUND

### 1.1 Description of the condition

Dementia is a syndrome in which there is deterioration in cognitive function beyond the usual consequences of biological aging (WHO‐Dementia, 2021). Patients with dementia often have obvious mental decline and show degeneration in cognitive, emotional, and communication functions (Arvanitakis, 2019). Current research has identified many causes of dementia, including primary neurological, medical conditions, and neuropsychiatric, with some cases involving multiple causes. Neurodegenerative dementias (such as Alzheimer's disease [AD] and Lewy body dementia) are most common in the aged, and traumatic brain injury and brain tumors are common in young people (Gale, 2018).

Dementia has become a considerable public health problem in many parts of the world because of its increasing incidence and long duration, with associated heavy burdens on patient caregivers and the cost of care (Livingston, 2020). AD is the most common form of dementia, with an incidence of about 20% among elderly people over 80 years old. Studies have shown that with the aging of the population, the number of patients with AD will continue to increase significantly (GBD 2019 Dementia Forecasting Collaborators, 2022). The World Alzheimer Report 2021 estimated that there were over 50 million people worldwide living with dementia in 2020. This number will almost double every 20 years, reaching 82 million in 2030 and 152 million in 2050. Much of the increase will be in developing countries. Presently, 60% of people with dementia live in low and middle‐income countries, but by 2050 this is predicted to rise to 71% (International Alzheimer's Disease, 2020). With huge social and family burdens, the annual total cost of providing routine services or treatment for each patient with dementia in the United States is as high as €42,898.65, while in Europe, the average annual total cost per patient with dementia is €32,506.73 (Cantarero‐Prieto, 2019).

Dementia is a progressive disorder. As dementia progresses, patients need more help and support in daily living. Many people in the early or middle stages remain largely independent, needing only minimal assistance with the activities of daily living (ADL). At this stage, it is important to focus on the person's capabilities and not perform all tasks for them. However, at the later stage, dementia has a severe impact on most aspects of a person's life, and eventually, the person will need full‐time help performing daily activities and personal care tasks, such as eating, washing, and dressing (Alzheimer's Society, 2020). Unlike some acute diseases, dementia is often not fatal. Early dementia patients only experience memory loss. However, over time, the patient's cognitive ability will decline significantly. Patients with severe dementia may become unable to carry out ADL and exhibit other psychological and physical problems. Although some drugs can delay the development of these adverse symptoms, there is no cure (WHO‐Dementia, 2021). Furthermore, the effectiveness of drug treatment is low while the cost is high, placing a large financial burden on patients, families, and society. The challenging behavior problems displayed by older adults with dementia present make it more difficult for personal and professional caregivers to provide care. These behaviors also interfere with effective communication. This situation reduces the patient's willingness to seek active treatment, which greatly impacts the quality of life (QOL) of the patients, further affecting the harmony of the family and social stability, and has become the focus of social attention (Moniz‐Cook, 2017; Oboudiyat, 2013). In these contexts, other models of care for providing better support for people with dementia are being studied (Callahan, 2017; Yang, 2018), and home support provided by a combination of formal services and informal caregivers may be more effective. This is also a goal of the National Dementia Strategy in England (Giebel, 2019).

### 1.2 Description of the intervention

Home‐based care (HBC) is defined as “care at the patient's residence to supplement or replace hospital care including medical management, palliative care, and social support” by the Committee on a National Strategy for AIDS (CNSA) (Wood, 2018). A similar definition is used by the World Health Organization (WHO) where community HBC is defined as any form of care given to ill people in their homes (Young, 2010). HBC refers to the various types of care, treatments, and support provided to patients in their homes, such as counseling and teaching, symptom management, and palliative care (Chen, 2017). It can be roughly divided into five categories: medicare skilled home health, informal services, formal personal care services, hospital‐at‐home services, and physician house calls (Landers, 2016). Many studies have indicated the need to provide home care for people with all diseases and thus, reduce the stigma associated with providing home care for only a particular disease (Young, 2010).

We are interested in formal HBC based on the definition of HBC. In this review, formal HBC is defined as receiving help from professional or paid workers rather than informal caregivers (i.e., most commonly family members and friends) in the home (Van Houtven, 2020). Home is defined as an individual's place of residence (e.g., apartments and residential homes) rather than special institutions (e.g., hospice, long‐term care facilities, and nursing homes).

### 1.3 How the intervention might work

HBC includes a wide range of services provided in the home rather than in a hospital or care community (Young, 2010). It can allow a person with dementia to stay in their own home, which also can greatly assist caregivers. Not all HBC is the same. Some HBC provides nonmedical help, such as assistance with ADL. Other types of HBC involve medical care delivered by a licensed health professional, such as a nurse or physical therapist. In this review, we only incorporated services provided by professional or paid workers, mainly for people with dementia through the following types:

1. Companion services: help with supervision, recreational activities, or providing companionship.
2. Personal care services: help with bathing, dressing, toileting, eating, exercising, or other personal care.
3. Homemaker services: help with housekeeping, shopping, or meal preparation.
4. Skilled care: help with wound care, injections, physical therapy, and other medical needs provided by a licensed health professional. A home health care agency often coordinates these types of skilled care services once they have been ordered by a physician.

### 1.4 Why it is important to do this review

Although some studies have shown that increased informal caregiving effectively reduced public healthcare spending by decreasing the amount of formal HBC delivered, the results were dependent upon the specific type of formal HBC (Bremer, 2017). As people age and dementia worsens, patients need more support at home, which includes a wider range and more professional services (GBD 2019 Dementia Forecasting Collaborators, 2022). The decreased availability of persons who can provide informal care and the increasing proportion of older people with dementia have increased the demand for formal care and services at home (Bökberg, 2015).

Previous reviews have explored the effects of HBC on people with dementia and family caregivers. However, conflicting results have been reported for some outcomes, such as anxiety and depression, and reviews on the effects of formal HBC and comparisons of all types of formal HBC are lacking. In 2015, Reilly (2015) evaluated the effectiveness of case management approaches to home support for people with dementia from the perspective of the different people involved compared to other forms of treatment. The research results showed that there was considerable heterogeneity between the interventions, outcomes measured, and time points across the included randomized clinical trials (RCTs), indicating the need to further investigate the effects of different types of interventions. Uncertain effects on patient depression, functional ability, and cognition have been reported, which also need further study. Almeida (2019) analyzed the effect of home‐based physical activity on people with dementia. The results showed that home‐based physical activity seemed to be safe and effective for improving some adverse patient symptoms while delaying declines in cognitive function and improving the burden on caregivers. Bennett (2019) published a systematic review (SR) on the effect of occupational therapy on patients with dementia and their family caregivers. This was a comprehensive research study of occupational therapy from multiple perspectives with various outcomes such as physiological, psychological, and economic burdens. The results showed that occupational therapy provided at home had a beneficial effect on dementia patients and their families. However, for some specific outcomes, such as anxiety and depression, the effects of occupational interventions are not yet clear and need to be studied further. Etxeberria (2021) analyzed the effectiveness of online support for the family caregivers of people with dementia, and the results showed that online support interventions were a valid resource for improving caregivers' psychological well‐being, with effects on depression, anxiety, burden, and caregiving competence. Chiao (2015) studied the factors constituting caregiver burden on the informal caregivers of people with dementia and found that caregiver sociodemographic factors and psychological factors were the two primary factors associated with caregiver burden. In addition, Lord (2019) focused on the influence of different HBC models on dementia patients at the theoretical model level. The study explored the theoretical models for providing better support for people with dementia in their homes. By including a total of 52 studies, a new evidence‐informed theoretical model was developed, incorporating the values and approaches integral to good quality dementia patient care, as well as care strategies and service models likely to deliver the quality care factors identified. The model can be used to design future interventions to enable people with dementia to live better and longer at home.

## 2 OBJECTIVES

The review will address the following questions:

1. Which formal HBC services for people with dementia have been tested in an RCT?
2. How effective are the different formal HBC services in improving the physical and mental health outcomes of people with dementia?
3. What is the cost and patient's health service usage of different formal HBC services?

## 3 METHODS

### 3.1 Criteria for considering studies for this review

#### 3.1.1 Types of studies

Randomized controlled trials (RCTs). We will include RCTs that the control group either received no intervention or received standard treatment. In addition, studies comparing two different types of formal HBC without a control group will also be included and analyzed separately. In this field, RCTs are technically and ethically feasible. Therefore, this review is limited to RCTs because they provide the best evidence of effectiveness.

#### 3.1.2 Types of participants

People of any age and either sex diagnosed with any type and stage of dementia.

We recognize that for some studies published earlier, the current diagnostic criteria for dementia may not be applicable to the participants. However, we believe that if the participants were diagnosed with dementia and used the acceptable criteria for the same period, this study can still be considered for inclusion in our review.

#### 3.1.3 Types of interventions

The interventions provided by paid professionals and teams in homes where people with dementia live will be included. Interventions provided by informal caregivers, families, and friends will be excluded. Studies set in a special institution (e.g., hospitals, nursing homes) or carried out through telemedicine or telecare will also be excluded.

#### 3.1.4 Types of outcome measures

If the study measured the effectiveness of formal HBC on at least one or more of the following outcomes, it will be included in this study.

##### 3.1.4.1 Primary outcomes

1. Changes in functional performance (e.g., measured by the Alzheimer's Disease Cooperative Study‐Activities of Daily Living [ADCS‐ADL] [Galasko, 1997]).
2. Changes in cognitive function (e.g., as measured by the Alzheimer's Disease Assessment Scale‐Cognitive subscale [ADAS‐Cog] [Rosen, 1984]).
3. Physical injuries (e.g., falls).
4. QOL (e.g., measured by the two‐item Quality of Life Scales [Graham, 1987]).
5. Mood: depression and anxiety (e.g., anxiety was measured by the Self‐Rating Anxiety Scale [SAS; Jegede, 1977] and depression was measured by the Self‐Rating Depression Scale [SDS; Zung, 1965]).
6. Death.

##### 3.1.4.2 Secondary outcomes

1. Caregiver burden (family/patients).
2. Acceptability of treatment (patients).
3. Cost of intervention.
4. Health service usage (patients).

### 3.2 Search methods for identification of studies

The pilot searches have been conducted, we will conduct a systematic search of all retrievable studies and reports, as well as hand‐search journals to determine the best available evidence. The reference lists from prior related reviews and included studies will be reviewed for potential studies. We will also conduct forward citation searching using Google Scholar to search for studies citing our included studies. At the same time, the search has no date, language, or location restrictions.

#### 3.2.1 Electronic searches

The Cochrane Database of Systematic Reviews and the Central Register of Controlled Trials (https://www.cochranelibrary.com/).

The Campbell Library (https://www.campbellcollaboration.org/).

PubMed (https://pubmed.ncbi.nlm.nih.gov/).

Embase (https://www.embase.com/).

Web of Science Core Collection (Web of Science).

PsycInfo and PsycArticles (EBSCOhost).

CINAHL (EBSCOhost).

China National Knowledge Infrastructure (https://www.cnki.net/).

VIP Chinese Science and Technique Journals Database (http://www.cqvip.com/).

The Chinese Biomedical Database (SinoMed).

Wanfang Data (https://www.wanfangdata.com.cn).

PubMed search strategy: Table 1.

**Table 1:** PubMed search strategy

#	Searches
#1	“Dementia”[Mesh] OR “Delirium”[Mesh] OR “Wernicke Encephalopathy”[Mesh] OR “Neurocognitive Disorders” [Mesh] OR “Dementia, Multi‐Infarct”[Mesh] OR “Dementia, Vascular”[Mesh] OR “Alzheimer Disease”[Mesh] OR “Lewy Body Disease”[Mesh] OR Delirium[Mesh] OR Huntington Disease[Mesh] OR “Pick Disease of the Brain”[Mesh] OR Kluver‐Bucy Syndrome[Mesh] OR Creutzfeldt‐Jakob Syndrome[Mesh]
#2	dement*[Title/Abstract] OR deliri*[Title/Abstract] OR alzheimer*[Title/Abstract] OR “organic brain disease”[Title/Abstract] OR “organic brain syndrome”[Title/Abstract] OR “benign senescent forgetfulness”[Title/Abstract] OR creutzfeldt[Title/Abstract] OR jcd[Title/Abstract] OR cjd[Title/Abstract] OR huntington*[Title/Abstract] OR binswanger*[Title/Abstract] OR korsako*[Title/Abstract] OR chronic cerebrovascular[Title/Abstract] OR supranuclear palsy[Title/Abstract] OR cerebr* deteriorat*[Title/Abstract] OR cerebral* insufficient*[Title/Abstract] OR pick* disease[Title/Abstract] OR mci[Title/Abstract] OR “subjective memory complaint”[Title/Abstract] OR “episodic memory”[Title/Abstract] OR “incipient dementia”[Title/Abstract] OR “pre‐clinical ad”[Title/Abstract] OR “pre‐clinical alzheimer*“[Title/Abstract] OR lewy*[Title/Abstract] OR Wernicke*[Title/Abstract]
#3	cognit*[Title/Abstract] OR memory*[Title/Abstract] OR mental*[Title/Abstract]
#4	declin*[Title/Abstract] OR impair*[Title/Abstract] OR los?[Title/Abstract] OR deteriorat*[Title/Abstract]
#5	#3 AND #4
#6	#1 OR #2 OR #5
#7	“Home Care Services”[Mesh] OR Community Health Nursing[Mesh] OR Social Support[Mesh] OR house calls[Mesh] OR “Home Care Agencies”[Mesh] OR “Home Health Nursing”[Mesh] OR “Intermediate Care Facilities”[Mesh] OR Patient Care Team[Mesh] OR Health Services for the Aged[Mesh]
#8	home‐based OR home based
#9	home[Title/Abstract] OR in‐home[Title/Abstract] OR at‐home[Title/Abstract] OR house[Title/Abstract] OR community[Title/Abstract] OR neighbo?rhood[Title/Abstract] OR family[Title/Abstract] OR lay[Title/Abstract] OR volition[Title/Abstract] OR non‐professional[Title/Abstract] OR nonprofessional[Title/Abstract] OR para‐professional[Title/Abstract] OR paraprofessional[Title/Abstract] OR professional[Title/Abstract] OR domiciliary[Title/Abstract] OR respite[Title/Abstract] OR residential[Title/Abstract] OR apartment[Title/Abstract] OR carer*[Title/Abstract] OR caregiver*[Title/Abstract] OR sta*[Title/Abstract] OR worker*[Title/Abstract] OR nurs*[Title/Abstract] OR health personnel[Title/Abstract] OR pharmacist[Title/Abstract] OR occupational[Title/Abstract] OR health care[Title/Abstract] OR patient care team*[Title/Abstract] OR patient care agenc*[Title/Abstract] OR patient care institute*[Title/Abstract] OR patient care compan*[Title/Abstract] OR “geriatric health service*“[Title/Abstract]
#10	visit*[Title/Abstract] OR support[Title/Abstract] OR program*[Title/Abstract] OR intervention[Title/Abstract] OR care*[Title/Abstract] OR management[Title/Abstract] OR service*[Title/Abstract] OR nursing[Title/Abstract]
#11	#9 AND #10
#12	#7 OR #8 OR #11
#13	randomized controlled trial[Publication Type] OR controlled clinical trial[Publication Type] OR randomized[Title/Abstract] OR placebo[Title/Abstract] OR clinical trials as topic[mesh: noexp] OR randomly[Title/Abstract] OR trial[Title]
#14	animals[Mesh] NOT humans[Mesh]
#15	#13 NOT #14
#16	#6 AND #12 AND #15

#### 3.2.2 Searching other resources

ClinicalTrials.gov (clinicaltrials.gov).

Grey Literature Report in 1999 to 2016 (www.greylit.org).

The International Clinical Trials Registry Platform of the World Health Organization (ICTRP, apps.who.int/trialsearch/Default.aspx).

Google Scholar (scholar.google.be).

Age UK (www.ageuk.org.uk/our-impact/policy-research/publications/).

Centre for Ageing Better (www.ageing-better.org.uk/publications).

International Longevity Centre UK (ILCUK, ilcuk.org.uk/reports/).

WHO Ageing and life‐course Program (www.who.int/ageing/data-research/en/).

National Ageing Research Institute (NARI) in Victoria, Australia (www.nari.net.au/publications/overview-about-publications).

UK Dementia Research Institute (https://ukdri.ac.uk/).

Alzheimer's Society (https://www.alzheimers.org.uk/).

Alzheimer's Association (https://www.alz.org/).

In addition, to ensure we identify the most recent references, we will hand‐search the last 5 years of key journals such as

Alzheimer's and Dementia (https://alz-journals.onlinelibrary.wiley.com/journal/15525279).

American Journal of Alzheimer's Disease & Other Dementias (https://journals.sagepub.com/home/aja#).

Dementia (https://journals.sagepub.com/home/dem).

Journal of Dementia (https://www.omicsonline.org/journal-dementia.php).

The final list of hand‐searched journals will be documented in the review.

### 3.3 Data collection and analysis

#### 3.3.1 Selection of studies

**Figure 1:**
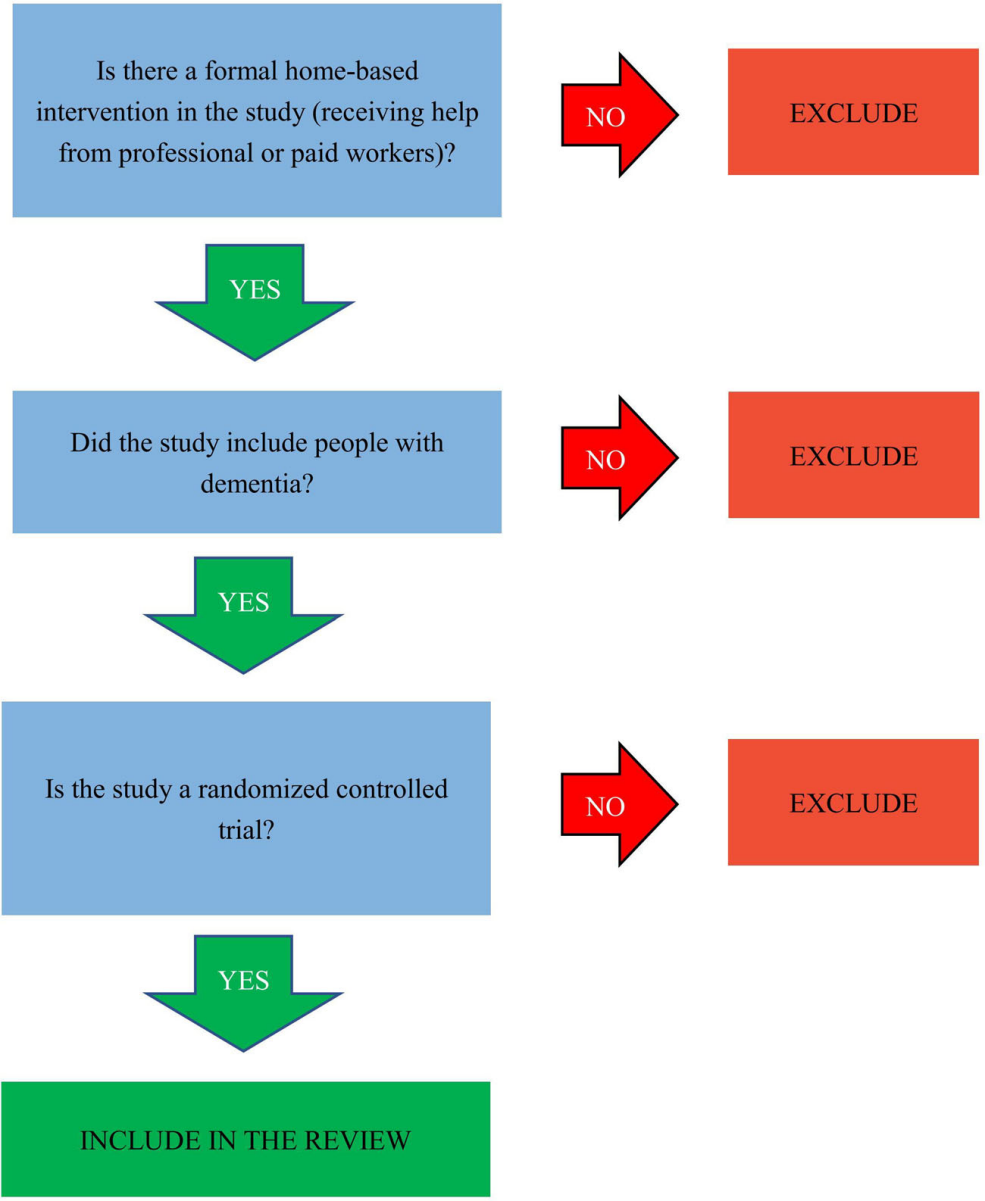
Screening tool

Two authors will independently conduct a rigorous trial screening (Figure 1). The entire screening process will be executed through EndNote X9 software and Rayyan QCRI. First, the duplication will be removed by two software, and then two authors will conduct preliminary screening based on the trial title and abstract. Second, for the initially included trials, the two authors will rescreen by searching the full text of these trials to determine the final included trials. In this process, when the opinions of the two reviewers differ, the differences will be resolved by consultation with a third author. At the same time, during the preliminary screening of the trial, if there is not enough information to exclude a study, the study will be further screened through the full text.

#### 3.3.2 Data extraction and management

Two authors will independently extract data based on the pre‐designed tables of this review using an Excel spreadsheet. Quantitative data will be entered into RevMan version 5.3 software and checked for accuracy. The extracted content mainly includes the following: the first author name, publication year, country, gender, and age characteristics of participants, sample size, study design, statistical analysis, intervention, and so on. Interventions (and comparison interventions) will be extracted where possible based on established domains (Campbell, 2018; Reilly, 2015): the name and type of intervention, the logic, mechanisms, or rationale of intervention, intervention materials, intervention goals, breadth of services spanned, roles and range of tasks, provider/delivery method of intervention, and intervention settings, intensity and duration of intervention, intervention adaptation (adaptation during implementation to respond to changing circumstances), intervention integrity/fidelity (degree to which the intervention was delivered according to original design), and any contextual factors that may shape implementation effectiveness (Supporting Information: Appendix 1).

#### 3.3.3 Assessment of risk of bias (RoB) in included studies

The RoB in the trials will be assessed by the tool recommended by the Cochrane Handbook Version 5.1.0 (Cochrane Collaboration; United Kingdom) according to the following seven aspects: random sequence generation, allocation concealment, blinding of participants and personnel, blinding of outcome assessment, incomplete outcome data, selective reporting, and other bias. Every item will be classified as yes (“low RoB”), no (“high RoB”), or unclear (“moderate RoB”). When the RoB of all seven components is defined as “low RoB,” the trial will be defined as the overall “low RoB.” At the same time, when one or more of the seven bias components is classified as high risk, the trial will be graded as “high RoB.” In other cases, the trial will be graded “unclear risk.” Disagreements in bias classification will be resolved by discussions among the two reviewers and, if necessary, through discussions with the authors.

#### 3.3.4 Measures of treatment effect

We will calculate dichotomous outcomes as risk ratios (RRs) with 95% confidence intervals (CIs). For continuous outcomes, mean difference (MD) will be used for studies reporting continuous data and similar outcome measures with a 95% CI. If outcome measures differ between studies the standardized mean difference (SMD) will be calculated to combine results across scales.

#### 3.3.5 Unit of analysis issues

We recognize that for some included studies that may have one or more interventions are evaluated in a single study. We will include each pair‐wise comparison separately. In cases of multiple intervention durations, we will analyze each outcome at each intervention duration separately. Comparable studies taking measures at an intervention duration will be analyzed together, grouped as follows: short‐term (less than 6 months), medium‐term (6 months to less than 12 months), and long‐term (12 months or more) (Reilly, 2015). For trials with more than two arms, we will split the “shared” group into two or more groups with smaller sample size and include two or more (reasonably independent) comparisons, as described in the Cochrane Handbook (Higgins, 2022). For cluster‐randomized trials where groups of people are allocated to interventions, we will assess these studies for unit of analysis errors (Welch, 2018). If there are unit of analysis errors (i.e., analysis at the level of the individual, without adjusting for clustering), we will inflate the standard deviation using the variance inflation factor for each intervention arm, using an intra‐cluster correlation coefficient (ICC) from a similar trial or from a database of ICCs. For dichotomous outcomes, we will use the methods in the Cochrane Handbook for Systematic Reviews of Interventions to adjust the numerator and denominator for unit of analysis errors (Higgins, 2022).

#### 3.3.6 Dealing with missing data

we will contact the study authors to supply any missing or unreported data. If data could not be obtained, we did not include the study in the meta‐analysis, and the extent to which the results or conclusions of the review might be affected by this will be assessed and discussed.

#### 3.3.7 Assessment of heterogeneity

Forest plots will be inspected to visually investigate overlap in the CIs for the results of the individual studies. In addition, we will use Dixon's *Q*‐test and the *I*‐squared (*I* ^2^) statistical tests to assess result heterogeneity. When the *P*‐value is less than (<) 0.05 and *I* ^2^ greater than (>) 50%, the result will be recognized as heterogeneous and subgroup and sensitivity analyses will be performed to explore possible reasons for heterogeneity.

#### 3.3.8 Assessment of reporting biases

We will perform funnel plots and visually examine the signs of asymmetry to investigate publication bias and use Egger's test as a formal test of publication bias when the number of the included studies on an outcome is more than 10 (*n* ≥ 10).

#### 3.3.9 Data synthesis

The meta‐analysis will be performed using RevMan version 5.3 software. We will pool results from clinically similar interventions. For dichotomous outcomes, the Mantel–Haenszel method will be used, and we will combine RRs with 95% CIs from included studies. For continuous outcomes, the Inverse‐Variance (I‐V) method will be used, and we will calculate MD, or SMD if studies measure the outcome on different assessment scales, with 95% CIs. We will assess whether to use random‐effects or fixed‐effects meta‐analysis given expected heterogeneity based on variations in intervention, treatment population, and so on. Subgroup analysis will be conducted to examine the effects of varying interventions and populations, as well as the heterogeneity of included studies. In case a quantitative synthesis is not possible, study findings will be synthesized narratively (Campbell, 2020; Yang, 2018). The data (e.g., events, sample size, and point estimates) will be presented with forest plot or table and grouped by different outcomes and comparisons. For each comparison and outcome, a description of the synthesis findings would be provided, making clear the direction of outcome of each study (Campbell, 2020).

#### 3.3.10 Subgroup analysis and investigation of heterogeneity

As Reilly (2015) suggested, there was considerable heterogeneity between the interventions, outcomes measured, and time points across the studies related to home support for people with dementia. If sufficient data are available, the subgroup analysis will be performed to assess effects and explore potential sources of heterogeneity by:

1. Formal HBC intervention. Variations in: the type of intervention, intervention goals, intervention materials, provider/delivery method of intervention, intensity of intervention, duration (grouped as follows: short‐term “less than 6 months,” medium‐term “6 months to less than 12 months,” and long‐term “12 months or more”), intervention settings, the logic, mechanisms, or rationale of intervention, breadth of services spanned, and roles and range of tasks will be included where possible.
2. People with dementia. Variations in: age, gender, and severity of cognitive impairment will be included where possible.
3. Study design and the RoB of included studies.

We will restrict subgroup analyses to outcomes that have at least two studies available. We will compare subgroups using the formal statistical test for subgroup differences in RevMan 5.3. We will interpret the results with caution.

#### 3.3.11 Sensitivity analysis

Sensitivity analysis will be utilized to determine whether the pooled effect sizes are stability across components of RoB by limiting the meta‐analysis to a subset of all studies included in the original meta‐analysis. For each domain of the RoB checklists, we will take sensitivity analysis into account and limit the analysis to studies with a low RoB. Additionally, we will perform “leave‐one‐study‐out” method sensitivity analysis to look into how each study affects effect size estimates (Higgins, 2022).

## CONTRIBUTIONS OF AUTHORS

- Content: Yanfei Li, Xiuxia Li, Rui Li, Nan Chen
- Systematic review methods: Xiuxia Li, Kehu Yang
- Statistical analysis: Meixuan Li, Yanfei Li, Rui Li
- Information retrieval: Yanfei Li, Xiuxia Li, Rui Li, Nan Chen

## DECLARATIONS OF INTEREST

All authors have no conflicts of interest.

## Supporting information

Supplementary Information
