# Supplementary material for: PROTOCOL: Home‐based care for people with dementia: A systematic review
Source: Campbell Syst Rev. 2022 Nov 2;18(4):e1285. doi: 10.1002/cl2.1285 (PMC9629276; doi:10.1002/cl2.1285)
Supplement: Supplementary file 1 — Supplementary Information [file CL2-18-e1285-s001.docx]

**Appendix 1. Screening tool**

# Sources of support

## Internal sources

- No sources of support provided

## External sources

- No sources of support provided

# Feedback

# Appendices

## 1 Appendix 1. Article coding and data extraction

| Publication details | Author (S) |  |
| --- | --- | --- |
|  | Year of publication |  |
| Population | Age Group | Includes <65 |
|  |  | Includes 65+ |
|  |  | Includes 75+ |
|  |  | Includes 85+ |
|  | Sex/Gender | 0-25% female included |
|  |  | 25-50% female included |
|  |  | 50-75% female included |
|  |  | 75-100% female included |
| WHO Regions | South-East Asia | |
|  | Western Pacific | |
|  | European | |
|  | African | |
|  | The Americas | |
|  | Eastern Mediterranean | |
| World Bank Classification by Income | Low-income economies | |
|  | Lower-middle income economies | |
|  | Upper-middle income economies | |
|  | High-income economies | |
| Intervention | The name and type of intervention | |
|  | The logic, mechanisms, or rationale of intervention | |
|  | Intervention materials | |
|  | Intervention goals | |
|  | The breadth of services spanned | |
|  | The roles and range of tasks | |
|  | The provider/delivery method of intervention | |
|  | Intervention settings | |
|  | Intervention adaptation (adaptation during implementation to respond to changing circumstances) | |
|  | Intervention integrity/fidelity (degree to which the intervention was delivered according to original design) | |
|  | Other (any contextual factors that may shape implementation effectiveness) | |
|  | Duration of intervention | Short‐term (less than 6 months) |
|  |  | Medium‐term (6 months to less than 12 months) |
|  |  | Long‐term (12 months or more) |
|  | Number of sessions per week | |
|  | Number of sessions per day | |
|  | Duration of each session | |
|  | Time between sessions | |
|  | Cost | Cost of intervention by professional or paid workers |
|  |  | Cost of intervention by others |
| Services provided by carer | Meals | |
|  | Medication | |
|  | Therapeutic services | |
|  | Physical activity | |
|  | Social interaction | |
|  | Shopping | |
|  | Trips outside of home | |
|  | Other | |
|  | Type of service | Companion services: help with supervision, recreational activities, or providing companionship |
|  |  | Personal care services: help with bathing, dressing, toileting, eating, exercising, or other personal care |
|  |  | Homemaker services: help with housekeeping, shopping, or meal preparation |
|  |  | Skilled care: help with wound care, injections, physical therapy, and other medical needs provided by a licensed health professional. A home health care agency often coordinates these types of skilled care services once they have been ordered by a physician |
|  |  | Other |
| Control | Usual Care | |
|  | No intervention | |
|  | Other | |
| Simple size | The number of participants in intervention group/control group | |
| Outcomes and related scales | Changes in functional performance, scales | |
|  | Changes in cognitive function, scales | |
|  | Physical injuries | |
|  | Quality of life, scales | |
|  | Depression, scales | |
|  | Anxiety, scales | |
|  | Caregiver burden (family/patients) | |
|  | Acceptability of treatment (patients) | |
|  | Cost of intervention | |
|  | Health service usage (patients) | |
| Quality assessment | High risk of bias | |
|  | Unclear risk of bias | |
|  | Low risk of bias | |
| Data for analysis | Quantitative information on per outcome (e.g., means, standard deviations, t‐values, simple size) (Note: all related outcomes will be extracted from the study and will be recorded in an Excel file for effect size calculations) | |
